# Site-Specific C-Terminal Fluorescent Labeling of Tau Protein

**DOI:** 10.1021/acsomega.2c06139

**Published:** 2022-12-12

**Authors:** Louise Bryan, Saurabh Awasthi, Yuanjie Li, Peter Niraj Nirmalraj, Sandor Balog, Jerry Yang, Michael Mayer

**Affiliations:** † Adolphe Merkle Institute, University of Fribourg, Chemin des Verdiers 4, CH-1700Fribourg, Switzerland; ‡ Transport at Nanoscale Interfaces Laboratory, Swiss Federal Laboratories for Materials Science and Technology, DübendorfCH-8600, Switzerland; § Department of Chemistry and Biochemistry, University of California San Diego, La Jolla, California92093-0358United States

## Abstract

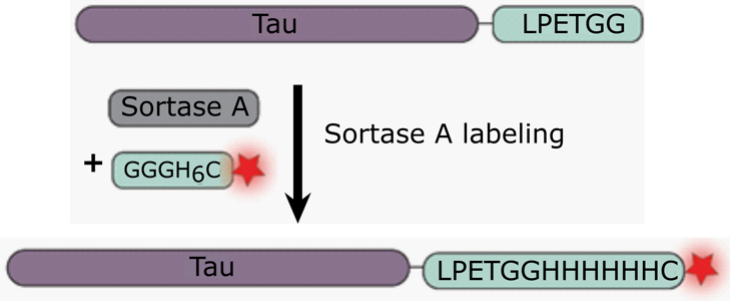

Formation of Tau protein aggregates in neurons is a pathological hallmark of several neurodegenerative diseases, including Alzheimer’s disease. Fluorescently labeled Tau protein is therefore useful to study the aggregation of these pathological proteins and to identify potential therapeutic targets. Conventionally, cysteine residues are used for labeling Tau proteins; however, the full-length Tau isoform contains two cysteine residues in the microtubule-binding region, which are implicated in Tau aggregation by forming intermolecular disulfide bonds. To prevent the fluorescent label from disturbing the microtubule binding region, we developed a strategy to fluorescently label Tau at its C-terminus while leaving cysteine residues unperturbed. We took advantage of a Sortase A-mediated transpeptidation approach to bind a short peptide (GGGH_6_-Alexa_647_) with a His-tag and a covalently attached Alexa 647 fluorophore to the C-terminus of Tau. This reaction relies on the presence of a Sortase recognition motif (LPXTG), which we attached to the C-terminus of recombinantly expressed Tau. We demonstrate that C-terminal modification of Tau protein results in no significant differences between the native and C-terminally labeled Tau monomer with regard to aggregation kinetics, secondary structure, and fibril morphology.

## Introduction

**Figure 1:**
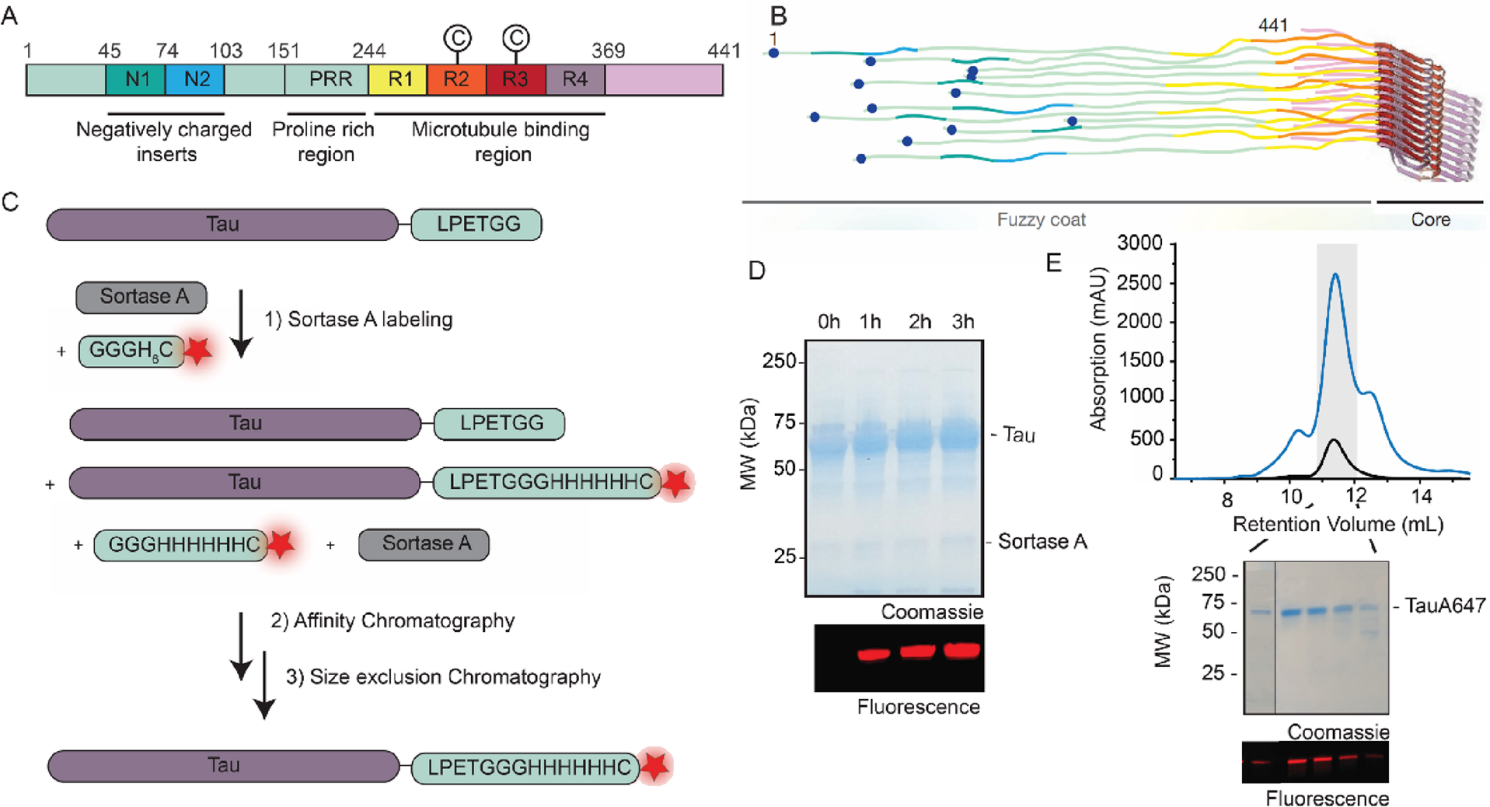
Full-length Tau protein and strategy for C-terminal labeling. (A) Schematic representation of full-length Tau protein (2N4R) colored by domain. N1, N2: N-terminal inserts, PRR: proline rich region, R1–R4: imperfect regions, and ⓒ indicates cysteine residues. (B) Schematic structure of Tau fibril consisting of a core and fuzzy coat. Reprinted with permission from ref (20). Copyright 2017 Springer Nature. (C) Schematic representation of C-terminal labeling of Tau protein with the enzyme Sortase A. The recombinantly expressed full-length Tau protein is extended on its C-terminus by addition of a -LPETGG peptide. Sortase A catalyzes the attachment of a His-tag with a covalently attached Alexa647 fluorophore. (D) Top: Coomassie blue staining of an SDS-PAGE gel of Tau protein after reaction for 0, 1 h, 2 h, and 3 h upon addition of Sortase A and labeled peptide; bottom: fluorescence image of the Tau band. (E) Size exclusion chromatography trace (FPLC) of Tau-Alexa647 purification (blue: absorbance at 214 nm, black: absorbance at 647 nm) and SDS-PAGE gel with top: Coomassie stain and bottom: fluorescence of Tau-Alexa647.

Tauopathies are a group of diseases affecting the brain caused by the aggregation of microtubule-associated protein Tau.^1,2^ Alzheimer’s disease (AD) is one of the most common neurodegenerative disorders, characterized by progressive memory loss and cognitive dysfunction, and Tau proteins are increasingly implicated in AD as well as other neurodegenerative diseases.^3,4^ Tau proteins are predominantly found in neuronal cells and are essential for the assembly and stabilization of microtubules.^5,6^ Human Tau protein exists in six different isoforms ranging in length from 352 to 441 amino acids that vary in the number of N-terminal inserts (N1 and N2) and contain three or four imperfect repeats (R1–R4). Repeats R1–R4 correspond to the microtubule-binding region (Figure 1A).^7,8^ The soluble monomeric form of Tau is thought to be intrinsically disordered and can exist in different conformations.^9,10^ Numerous post-translational modifications of Tau protein, such as phosphorylation, acetylation, or methylation, regulate its interactions with microtubules or other proteins.^11,12^ Phosphorylation is one of the most prevalent modifications with up to 85 potential sites on serine, threonine, or tyrosine residues.^13^ Hyper-phosphorylation of Tau results in impaired microtubule binding and leads to misfolding and aggregation, affecting neuronal stability and causing neuronal loss.^14,15^ The exact aggregation mechanism of Tau and hyper-phosphorylated Tau is still under investigation; however, it is thought to undergo a nucleation-dependent pathway.^16^ Studies have suggested that Tau also undergoes liquid–liquid phase separation, a property of intrinsically disordered proteins.^17^ The most toxic species are early aggregates of Tau protein, i.e., soluble or low-*n* oligomers.^18,19^ These small aggregates self-assemble into fibrils and finally into neurofibrillary tangles.^2^ High-resolution cryo-electron microscopy (cryo-EM) images of purified Tau aggregates from the brains of AD patients display an ordered core of pairs of protofilaments comprising regions R3 and R4 (Figure 1B), with the disordered N- and C-termini forming a so-called fuzzy coat.^20,21^

Single-molecule fluorescence microscopy has the potential to improve our understanding of Tau aggregation. Labeling of Tau protein with a high-quality fluorophore such as the Alexa647 dye used here is essential for such studies. It is, however, important to consider two key factors when designing a method to label Tau protein: First, the presence of two hydrophobic hexapeptide sequences, _275_VQIINK_280_ (known as PHF6*) in R2 and especially _306_VQIVYK_311_ (PHF6) in R3,^22^ which are strongly associated with Tau protein aggregation and should not be perturbed.^22,23^ Second, the presence of two cysteine residues (at positions 291 and 322) (Figure 1A), which are involved in intermolecular disulfide bond formation, should be preserved. In particular, Cys-322 is considered to drive the initial dimerization of Tau protein monomers and aggregation into paired helical filaments.^24−26^ Since modification of these cysteine residues with a fluorophore may influence the aggregation mechanism of Tau protein,^27^ we sought a strategy to label the protein on either the N- or C- terminus. Both termini are part of the fuzzy coat and are located away from the core region of Tau fibrils (Figure 1B). To our knowledge, labeled Tau has so far only been prepared using K_18_ or shorter Tau isoforms by modification of amine groups or by site-specific mutagenesis of one of the two cysteine residues in the core region of full-length Tau.^28−32^

Here, we employed a site-specific approach to label the C-terminal end of full-length Tau protein and investigated the effects of modifications on its secondary structure and aggregation kinetics compared to those of native Tau protein *in vitro*. We monitored the aggregation of native and fluorescently labeled Tau over time in the presence of the polyanionic inducer heparin^33^ using a ThT assay.^33,34^ In addition, we compared the morphology of the resulting Tau fibrils using transmission electron microscopy (TEM) and atomic force microscopy (AFM).^35−38^ These studies revealed that the native and C-terminally labeled Tau protein exhibit similar properties with respect to the secondary structure, fibril morphology, and aggregation kinetics.

## Results and Discussion

### C-Terminal Labeling of Tau Protein

We explored a Sortase A-mediated labeling strategy to fluorescently label Tau protein while causing minimal perturbation at the core region of the protein in the fibrillar state.^39−41^ The method relies on the addition of a specific recognition motif LPXTG (X being any amino acid) to the C terminus of recombinantly expressed Tau protein. Sortase A from *Staphylococcus aureus* cleaves off the terminal glycine and forms a thioester with threonine. This step is followed by conjugation to a (G)_*n*_ peptide/protein. We thereby used full-length Tau with the addition of the short sequence LPETGG at its C-terminus and enzymatically ligated it to a short peptide bearing a His-tag and a covalently attached fluorescent molecule: GGGH_6_C-Alexa647 (Figure 1C). We chose Alexa647 since it is a high-quality, photostable, far-red fluorescent dye and to prevent a spectral overlap with Thioflavin T whose fluorescence can be excited from 385 to 450 nm and whose emission maximum ranges from 445 to 482 nm.^42^ We added the hexahistidine sequence to the peptide to provide an additional tag for efficient separation of the labeled from the unlabeled protein and to concentrate labeled Tau species since the Sortase A labeling reaction typically provides labeling efficiencies of 20 to 90%, largely dependent on the Sortase A variant.^43^ We used the commercially available Sortase pentamutant (Sortase 5A, with a His-tag), which increases the rate and efficiency of Sortase labeling compared to the wild-type Sortase A.^44^ The labeling reaction was carried out at 10 °C to prevent degradation of the Tau protein or the formation of small aggregates. The reaction mixture was then purified on a Ni-NTA column where the labeled protein, GGGH_6_-Alexa647, and Sortase A, all containing His-tags, were retained on the column. This affinity chromatography was followed by size exclusion chromatography to purify the labeled protein from the remaining molecules in the mixture (Figure 1D,E). This procedure purified C-terminal labeled Tau protein for further structural characterization of the monomeric form of Tau and its aggregates.

### Secondary Structure and Aggregation Kinetics of Labeled and Native Full-Length Tau

**Figure 2:**
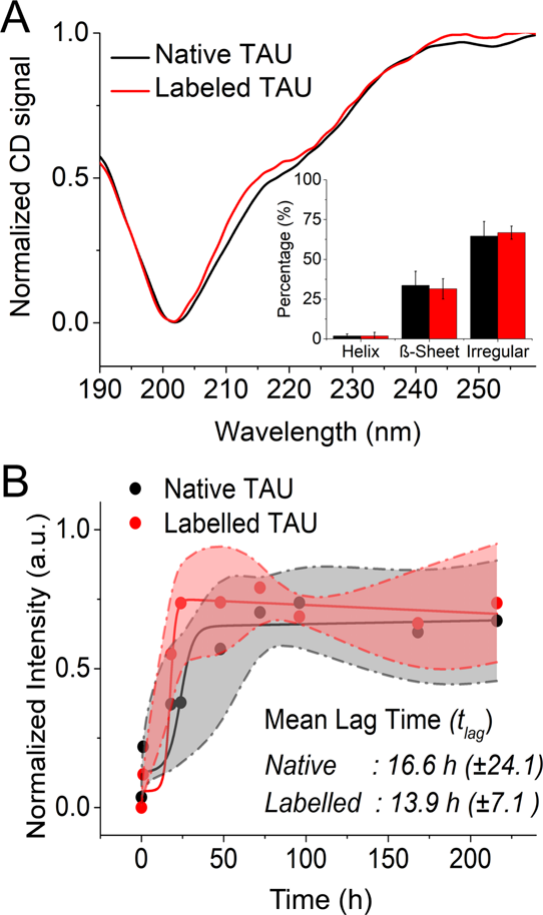
Comparison of the monomer secondary structure of native and labeled Tau in solution as well as their aggregation kinetics. (A) CD spectra of native (black) and labeled Tau (red) in their predominantly monomeric form. The inset shows the percentage of different secondary structures for native and labeled Tau as determined using the online server BeStSel.^45^ (B) ThT fluorescence intensity measurements over time to compare the aggregation kinetics of native and labeled Tau protein with excitation and emission wavelengths of 412 and 490 nm, respectively. The data are the average of three independent measurements. The shaded regions in gray and red represent the 95% confidence interval for the native and labeled Tau protein, respectively.

We determined the secondary structure of monomeric Tau-A647 and native full-length Tau protein by CD spectroscopy. Figure 2A shows a minimum at 200 nm for both native Tau and Tau-A647, indicating that they are unstructured in solution and the addition of a short peptide sequence with a fluorescent dye on the C-terminus did not change the secondary structure. Furthermore, we analyzed the CD spectra and determined the propensity of different secondary structural elements such as α-helix, β-sheet, and irregular or disordered state for native and labeled Tau protein monomers. Table 1 reveals a similar secondary structure in both the variants (i.e., native and labeled), suggesting a minimal effect of C-terminal labeling on the Tau protein structure in solution (Figure 2A). Statistical analysis confirmed no significant difference in the secondary structure of native and labeled Tau protein (see Figure S1).

**Table 1:** Fraction of Different Secondary Structures for Native and Labeled Tau Protein

protein variant	α-helix (%)	β-sheet (%)	irregular (%)
native Tau	1.9 ± 1.2	33.6 ± 8.9	64.6 ± 9.2
labeled Tau	1.8 ± 2.3	31.5 ± 6.3	66.7 ± 4.1

We also carried out a comparative analysis of aggregation kinetics of labeled and native full-length Tau. To follow their aggregation, each Tau variant was incubated with the inducer, i.e., heparin (using a molar ratio of Tau:heparin of 4:1), and ThT at 37 °C in Tau aggregation buffer and their fluorescence spectrum was measured at regular time points (Figure 2B). While the variability between repeat experiments was considerable due to the stochastic aspect of these aggregation assays, we observed overall a similar trend in the case of native and labeled Tau protein with the ThT fluorescence increasing overtime corresponding to the formation of aggregated species. The analysis of aggregation kinetics revealed that the duration of the lag phase, *t*_lag_, was 16.6 h for native Tau and 13.9 h for labeled Tau. Given the variations and corresponding uncertainty in the half time (*t*_1/2_) and lag time (*t*_lag_) (see Figure S3 and Table S2), these parameters were not significantly different between native and labeled Tau protein. Statistical analysis revealed no significant difference in the aggregation kinetics of native and labeled Tau protein (see Figure S2 and Table S1).

### Labeled and Native Tau Exhibit Similar Fibril Morphology

**Figure 3:**
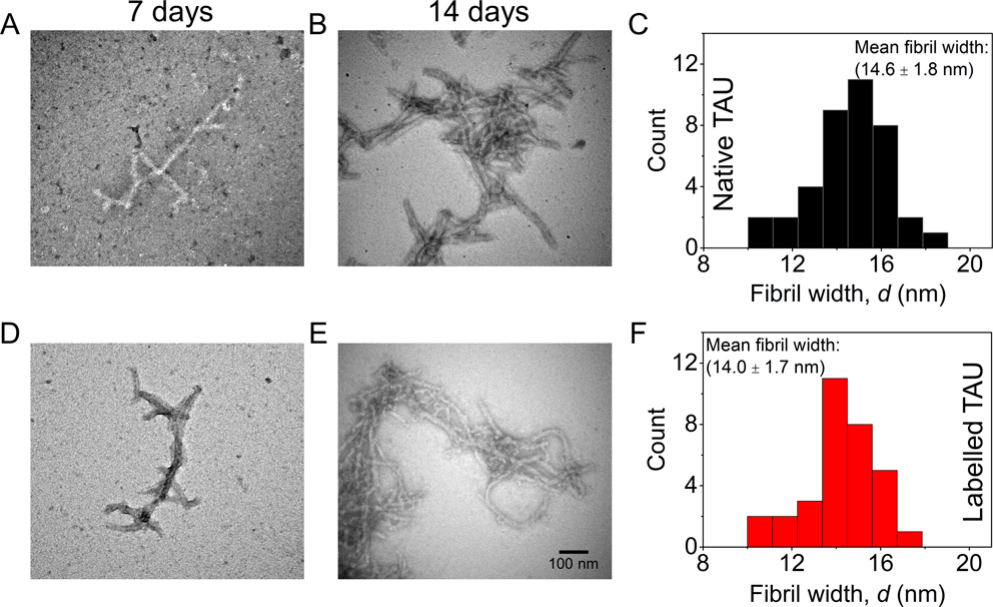
Transmission electron microscopy (TEM) imaging and characterization of fibril width. (A) and (B) TEM images of native Tau protein aggregates incubated for 7 and 14 days at 37 °C under shaking conditions in the presence of heparin. (D) and (E) TEM images of Alexa_647_-labeled Tau protein incubated for 7 and 14 days, respectively. (Scale bar: 100 nm). (C,F) Histograms of the fibril width after 14 days of aggregation. Statistical analysis revealed no significant difference in the fibril width of native and labeled Tau protein.

To compare the fibril morphology, we carried out TEM and AFM imaging of aggregates formed by native and labeled full-length Tau protein. Figure 3 shows that TEM imaging of Tau fibrils after 7 and 14 days of aggregation *in vitro* induced by heparin revealed similar fibril morphology. From these images, we determined the width of fibrillar aggregates. Both the native full length-Tau protein and labeled Tau-A647 exhibit similar fibril widths of 14.6 ± 1.8 and 14.0 ± 1.7 nm, respectively (Figure 3C,F). Earlier studies have shown that Tau fibrils extracted from brain samples exhibited a width of 19 nm, whereas *in vitro* heparin-induced Tau protein fibrils exhibited a width of 16.9 nm.^46^ More recently, Zhang *et al.* showed that heparin-induced Tau fibrils are in fact highly heterogeneous with a width ranging from 4 to 25 nm.^47^ The estimates of fibrillar width for both the native and labeled Tau protein determined here are in good agreement with these earlier reports.

**Figure 4:**
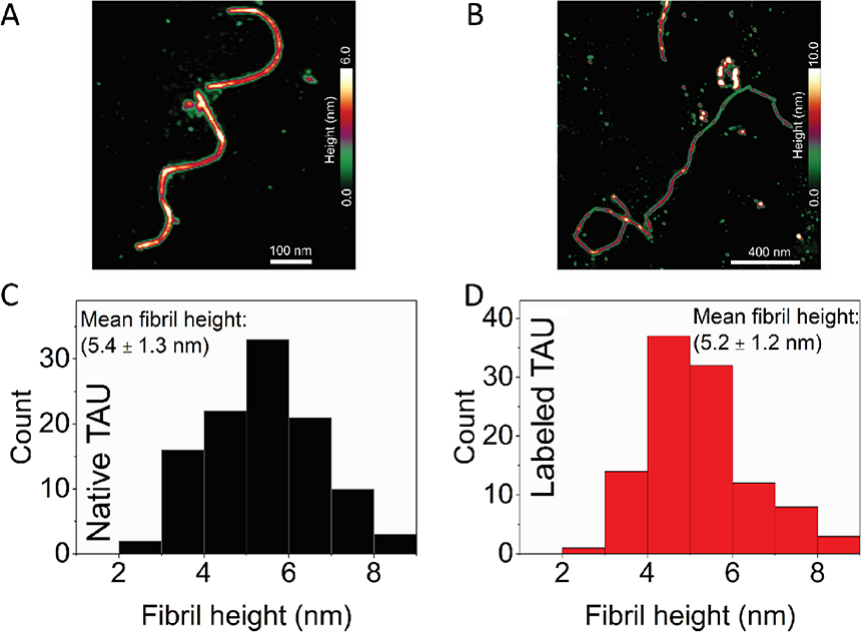
Atomic force microscopy (AFM) imaging and characterization of the height of Tau fibrils. AFM image showing the topography of (A) native and (B) labeled Tau fibrils after 7 days of incubation. Height distribution (based on individual cross-sectional analyses of the AFM data) obtained on (C) native and (D) labeled Tau fibrils. A mean fibril height of 5.0 ± 1.1 nm was obtained for the native Tau fibrils, and a mean fibril height of 5.6 ± 1.2 nm was calculated for the labeled Tau fibrils from the height distribution plots. Statistical analysis revealed no significant difference in the fibril height of native and labeled Tau protein.

Furthermore, we characterized the fibrils formed after 7 days of incubation by labeled and native Tau protein using liquid-based AFM to estimate their height (Figure 4A–F). Histograms of the height of individual fibrils revealed a range of 5.0 ± 1.1 nm for native Tau and 5.6 ± 1.2 nm for Tau-A647 (Figure 4F). Overall, the high-resolution AFM images didnot show significant differences in topography, morphological variations along the fibril, height profile, or alignment between the native and labeled Tau fibrils.

Earlier studies have demonstrated possibilities to use Sortase A-mediated labeling for *in vivo* protein labeling. Sortase-mediated ligation of LPETG-tagged proteins is possible *in vivo* when expressed in *Caenorhabditis elegans*.^48^ Co-expression of Sortase-A with the protein of interest *in vivo* has been used to achieve protein labeling, resulting in large quantities of labeled protein.^49^ Therefore, as an outlook, the site-specific C-terminal labeling approach of Tau protein demonstrated here may be useful to study Tau protein aggregation *in vivo* to improve our understanding of AD pathology.

## Conclusions

Using Sortase A-mediated labeling, this work demonstrates that (1) C-terminal labeling of Tau protein has minimal effect on secondary structure in solution, (2) both the labeled (i.e., Tau-647) and native-full length Tau protein exhibit similar aggregation kinetics, and (3) both the labeled and native Tau protein exhibit similar fibrillar morphology. We propose that the C-terminal labeling strategy of Tau protein presented here may therefore be useful for studies of Tau aggregation using (single-molecule) fluorescence methods with minimal effects on the structure of the native protein conformation.

## Materials and Methods

### Tau Labeling

The pET30a plasmid containing GST-Tau-LPETGG-H_6_ was synthesized by GenScript. The protein was expressed by Oscar Vadas at the Protein and Peptides Platform, University of Geneva, in *Escherichia coli* followed by purification. For labeling, 50 μM purified Tau-LPETGG was mixed with 150 μM peptide GGGH_6_-Alexa647 (Bio-Synthesis Inc.) and 3 μM Sortase A5 (Active Motif) in 20 mM Tris, 150 mM NaCl, 10 mM CaCl_2_, and 1 mM DTT, pH 7.4. The mixture was incubated at 10 °C for 3 h and then loaded onto a His Spintrap column (GE Healthcare). After 1 h incubation, the column was washed with 10 mM Tris, pH 7.8,100 mM NaCl, and 10 mM imidazole followed by elution with 10 mM Tris, pH 7.8, 100 mM NaCl, and 250 mM imidazole. The elute was concentrated using a Vivaspin 500, 3000 MWCO centrifugal concentrator (Sartorius, Germany) and then purified on a Superdex200 increase 10/300 column (GE Healthcare) in 10 mM Tris, 100 mM NaCl, and 1 mM DTT, pH 7.4, using an FPLC AKTA system (GE Healthcare). The fractions containing the purified labeled Tau protein were pooled, concentrated with a Vivaspin 500, 3000 MWCO centrifugal concentrator, aliquoted, flash frozen in liquid N_2_, and stored at −80 °C. At each step, samples were taken, mixed with SDS-PAGE loading buffer, and loaded onto an SDS-polyacrylamide gel for a 40 min run at 150 V to verify the presence of the protein.

### ThT Assays

The aggregation of native full-length Tau procured from Eurogentec and Tau-LPETGGGH_6_C-Alexa_647_ henceforth called Tau-A647 was monitored by measuring the fluorescence spectra of ThT over time using a fluorometer from Horiba Scientific. For the Tau aggregation assay, a solution containing the final concentration of 2 μM Tau, 0.5 μM heparin sodium salt (MW: 6000–30,000 g/mol) and 1 μM ThT was mixed in Tau aggregation buffer (25 mM sodium phosphate, 25 mM NaCl, 2.5 mM EDTA, 0.33 mM DTT, pH 6.8). The aggregation mix was incubated at 37 °C under constant agitation at 300 rpm in protein Lobind tubes (Eppendorf). Fluorescence emission spectra were measured at different time points (0, 1, 24, 48, 72, 96, 168, 192, and 216 h) by using an excitation wavelength of 412 nm and a slit of 5 nm in black quartz cuvettes (Hellma). The normalized ThT fluorescence intensity values plotted in Figure 2B are the ones at 490 nm wavelength. We estimate the lag time (*t*_lag_) of aggregation using eq 1:^50^
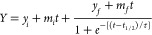
1where *Y* is the fluorescence intensity as a function of time *t*, *y_i_* and *y_f_* are the intercepts of the initial and final fluorescence values at the *y*-axis, *m_i_* and *m_f_* are the slopes of the initial and final baselines, *t*_1/2_ is the time needed to reach halfway through the elongation phase, and τ is the elongation time constant. The apparent rate constant, *k*_app_, for the growth of fibrils is given by 1/τ, and the lag time is usually defined as *t*_lag_ = *t*_1/2_ – 2τ.^50^

### CD Spectroscopy

CD spectra were acquired on a Jasco J-810 CD spectropolarimeter using 0.1 cm quartz cuvettes (Hellma) operated at 20 °C. To minimize buffer absorption, Tau samples with 1 to 2.5 μM concentration were dialysed overnight against 10 mM sodium phosphate buffer, pH 7.8, in Slide-A-Lyzer MINI dialysis devices (100 μL). CD spectra were recorded from 190 to 260 nm at a scan speed of 20 nm/min and an increment of 1 nm. Four scans were recorded for each sample. The buffer was used as a blank for background subtraction.

### AFM

A total of 5 μL solution containing fibrils of native Tau or labeled Tau (Tau-LPETGGGH_6_C-Alexa_647_) both at an equivalent monomer concentration of 100 nM in a buffer containing HEPES pH 7.4 was pipetted onto Si(111), an *n*-type substrate. The sample was left to settle for 30 min followed by injecting 10 μL of pure water to rinse the buffer salt solution. Atomic force microscopy images were acquired in tapping mode in pure water with a Bruker Multimode 8, E-scanner. The tip used was a Scanasyst-AIR, 0.4 N/m, 70 kHz (Bruker AFM probes). Images were recorded at a resolution of 1024 by 1024 pixels at a scan rate of 1 Hz. Analysis of AFM data was conducted using Bruker Nanoscope Analysis software.

### Electron Microscopy

Transmission electron microscopy images were recorded with a FEI Tecnai Spirit operating at 120 kV. Carbon-coated 300-mesh copper grids (Electron Microscopy Sciences, Hatfield) were plasma-cleaned for 5 s using an oxygen plasma cleaner (Zepto RIE, Dienner), and 5 μL of Tau protein (2 μM monomer concentration) sample in aggregation buffer consisting of 25 mM sodium phosphate, 25 mM NaCl, 2.5 mM EDTA, and 0.33 mM DTT, pH 6.8, was pipetted on top of the grids after 100 times dilution in water and incubated for 2 min. The grids were then washed with a water droplet. Uranyl acetate (2% w/v) was added (3 μL) and incubated for 2 min. Excess stain was blotted off with a filter paper and dried. We used ImageJ to estimate the width of Tau protein fibrils.^51^

## Supporting Information Available

Detailed information about statistical analysis (Supplementary Note 1), statistical analysis of results from secondary structure estimates (Figure S1), statistical analysis of results of aggregation kinetics, fibril diameter, and fibril height measurements (Figure S2), ThT fluorescence intensity measurements of native and labeled Tau protein (Figure S3), statistical analysis of ThT fluorescence intensity results (Table S1), and half time (*t*_1/2_), lag time (*t*_lag_), and elongation time constant (τ) values for the aggregation kinetics experiment of native and labeled Tau protein (Table S2) (PDF)
