# Supplementary material for: Site-Specific C-Terminal Fluorescent Labeling of Tau Protein
Source: ACS Omega. 2022 Dec 12;7(50):47009–14. doi: 10.1021/acsomega.2c06139 (PMC9773802; doi:10.1021/acsomega.2c06139)
Supplement: Supplementary file 1 — ao2c06139_si_001.pdf [file ao2c06139_si_001.pdf]

Site-specific C-terminal fluorescent labeling of Tau protein

Louise Bryan[1], Saurabh Awasthi[1$], Yuanjie Li[1], Peter Niraj Nirmalraj[2], Sandor Balog[1], Jerry Yang[3], Michael Mayer[1$]

[1] Adolphe Merkle Institute, University of Fribourg, Chemin des Verdiers 4, CH-1700 Fribourg, Switzerland

[2] Transport at Nanoscale Interfaces Laboratory, Swiss Federal Laboratories for Materials Science and Technology, Dübendorf CH-8600, Switzerland.

[3] Department of Chemistry and Biochemistry, University of California San Diego, La Jolla, CA, 92093-0358 USA

[$] Corresponding Author 1: michael.mayer@unifr.ch

[$] Corresponding Author 2: saurabh.awasthi@unifr.ch

Supplementary Note: Statistical analysis

We performed statistical hypothesis tests to determine whether the data contradict the claim that fluorescent labeling of Tau protein does not alter the aggregation kinetics, secondary structure, and aggregate morphology. The results shown in **Figure 2B**, **Figure 3C** and **3F**, and **Figure 4C** and **4D** (in the main text) were subjected to the Kruskal-Wallis test.[1] The Kruskal-Wallis test does not assume normality in the data, and essentially performs a one-way analysis of variance on the ranks of the data, and it may be used to decide whether or not there is a statistically significant difference between the medians of independent populations. One population (in the context of statistical testing) is the native Tau protein, and the other is the C-terminally labeled Tau protein. Based on the data, the test calculates a p-value corresponding to the null hypothesis (H_0). The null hypothesis is that the two populations represented by the respective data are not significantly different. This p-value estimates the probability of observing the results of the null hypothesis. We set the significance level to 0.05. If the p-value is less than the significance level, we reject the null

hypothesis. A p-value higher than the significance level indicates that there is no evidence in the data to reject the null hypothesis.

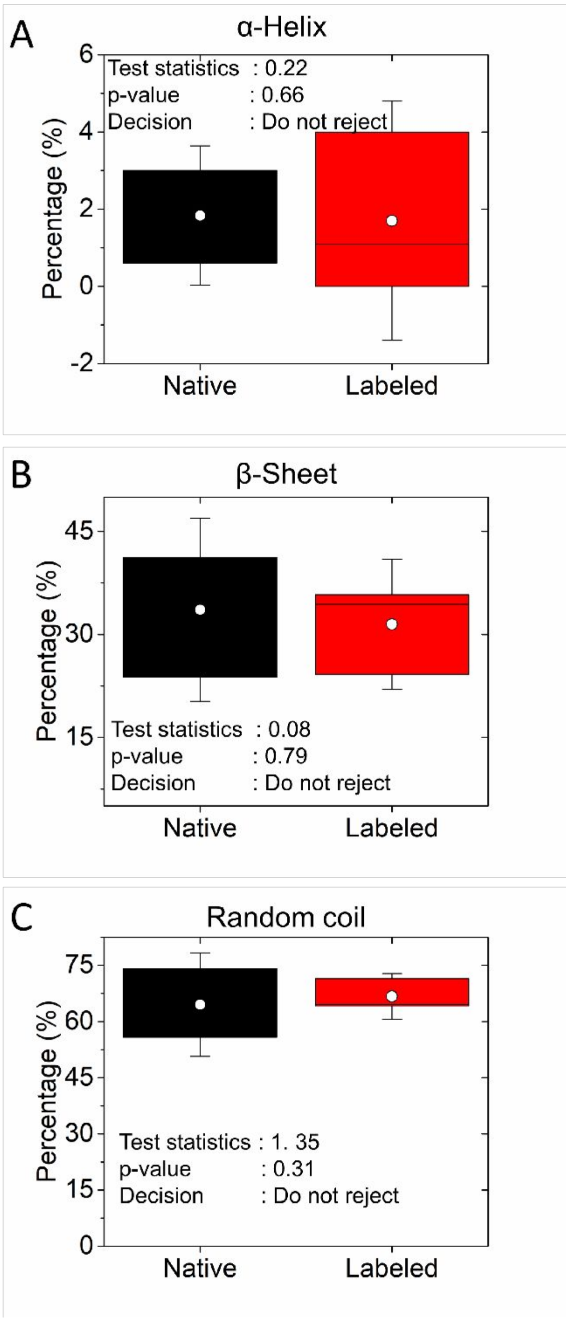

Figure. S1 Statistical analysis of results from secondary structure estimates using circular dichroism. Comparative analysis of different secondary structures i.e., α-helix **(A)** β-sheet **(B)**, ramdom coil **(C)** of native (black) and labeled (red) Tau protein monomer as determined by CD spectroscopy respectively. Statistical analysis revealed no significant difference in the secondary structures of native and labeled Tau protein. The horizontal lines in the box are representing the median and quartile values. The mean values are shown as open circles for different datasets, with the whiskers showing standard deviation (SD).

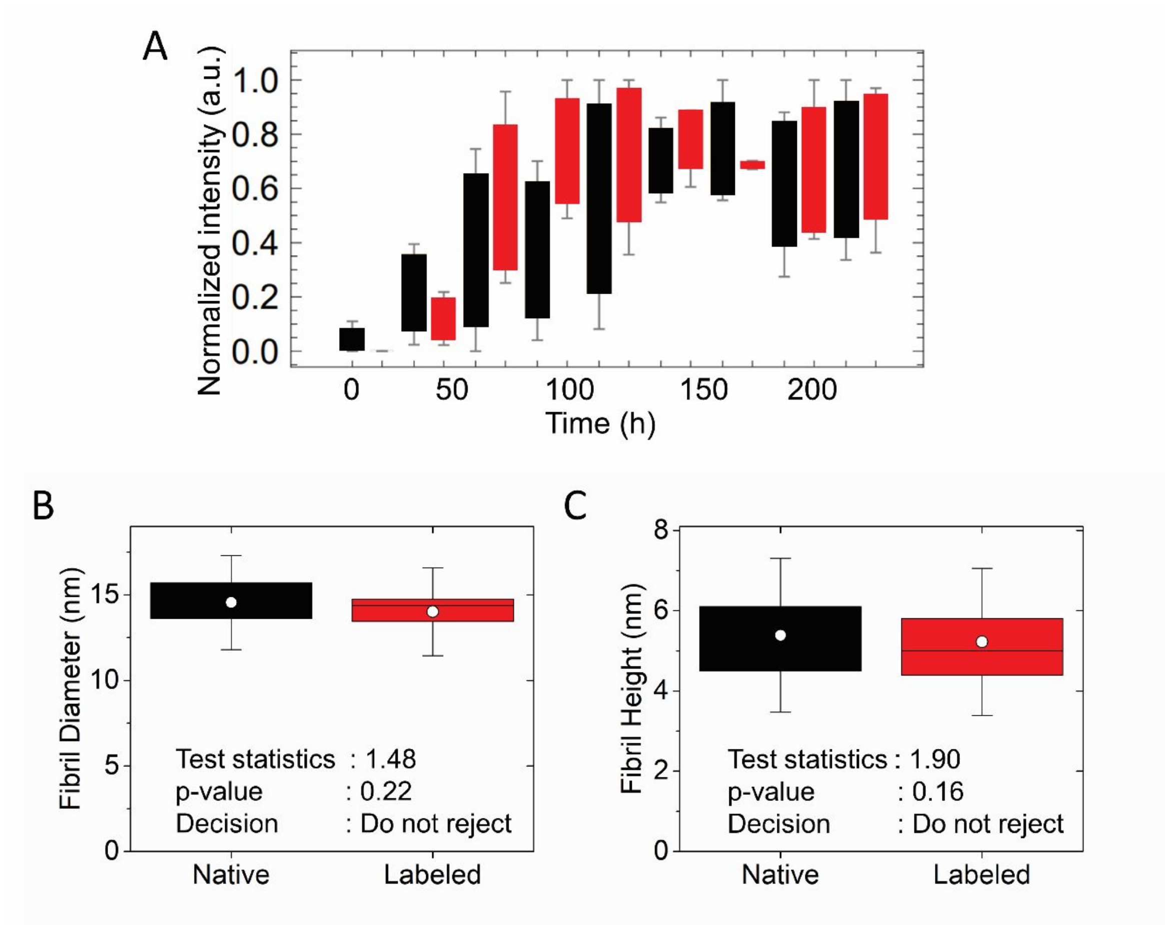

Figure. S2 Statistical analysis of results from aggregation kinetics, fibril diameter, and fibril height measurements. Comparative analysis of aggregation kinetics by normalized ThT fluorescence intensity **(A)** fibril diameter **(B)**, and fibril height **(C)** of native (black) and labeled (red) Tau protein as determined by TEM and AFM respectively. Overall, the results showed no significant differences between the native and C-terminally labeled Tau monomer with regard to fibril diameter, fibril height, and aggregation kinetics. The horizontal lines in the box are representing the median and quartile values. The mean values are shown as open circles for different datasets, with the whiskers showing standard deviation (SD).

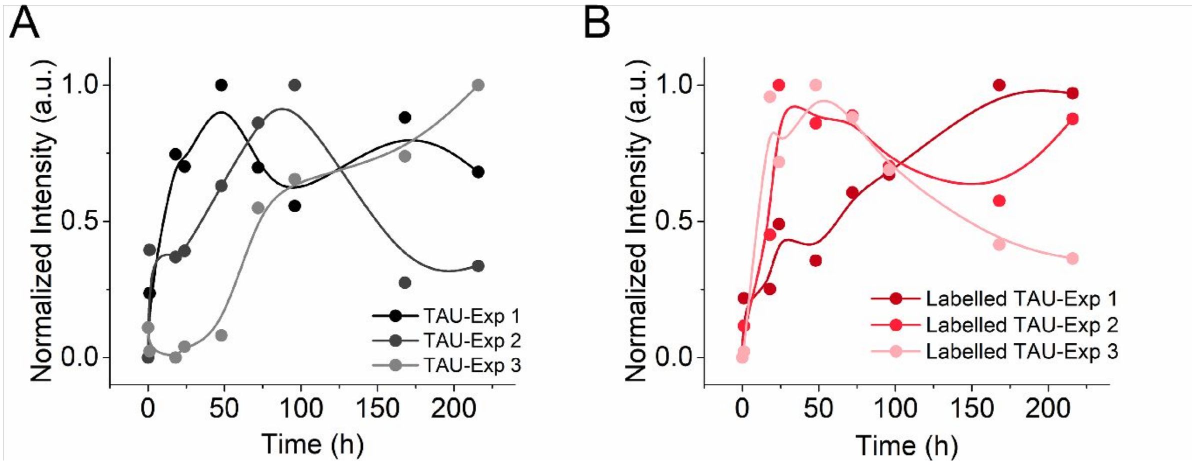

Figure. S3 ThT fluorescence intensity measurements of native (A) and labeled Tau protein (B) with excitation and emission wavelength of 412 nm and 490 nm respectively. The data points shown here the results of three independent experiments, due to the stochastic aspect of aggregation, the variability between repeat experiments is considerable.

Table. S1 Statistical analysis of ThT fluorescence intensity results from aggregation kinetics of native and labeled Tau protein.

Time (h)	Test Statistic	p-value	Decision
0	1	0.37	Do not reject the null hypothesis.
1	0.67	0.46	
18	0.36	0.58	
24	2.20	0.21	
48	0.26	0.64	
72	0.47	0.52	
96	0.43	0.57	
168	0.01	0.90	
216	0.05	0.82	

Kruskal-Wallis test[1] revealed no significant differences between the native and C-terminally labeled Tau protein with regard to their aggregation kinetics.

Table. S2 Half time ($t_{1/2}$), lag time (t_{lag}) and elongation time constant (τ) values for the aggregation kinetics experiment of native and labeled Tau protein.

Experiment	Native TAU			Labeled TAU		
	$T_{1/2}$	Lag time (t_{lag})	τ	$T_{1/2}$	Lag time (t_{lag})	τ
1	1.28	0.62	0.30	20.26	12.26	4
2	0.98	0.82	0.08	18.12	16.12	1
3	53.56	44.88	4.34	8.70	8.58	0.06

References

(1) In *The Concise Encyclopedia of Statistics*; Springer New York: New York, NY, 2008, pp 288-290.
